# Exergy Analysis of Phase-Change Heat-Storage Coupled Solar Heat Pump Heating System

**DOI:** 10.3390/ma14195552

**Published:** 2021-09-24

**Authors:** Chuanhui Zhu, Shubin Yan, Xiaodong Dong, Wei Zhang, Biyi Huang, Yang Cui

**Affiliations:** 1 The College of Electrical Engineering, Zhejiang University of Water Resources and Electric Power, Hangzhou 310018, China; yanshb@zjweu.edu.cn (S.Y.); zhangw@zjweu.edu.cn (W.Z.); huangby@zjweu.edu.cn (B.H.); ycui_ma13@hrbust.edu.cn (Y.C.); 2 Zhejiang-Belarus Joint Laboratory of Intelligent Equipment and System for Water Conservancy and Hydropower Safety Monitoring, Hangzhou 310018, China; 3 Tibet Autonomous Region Energy Research Demonstration Center, Lhasa 850000, China; nyzx_dod@sti.xizang.gov.cn

**Keywords:** phase-change heat-storage tank, solar collector, heat pump, heating, exergy analysis

## Abstract

With the rapid development of industrialization, the excessive use of fossil fuels has caused problems such as increased greenhouse gas emissions and energy shortages. The development and use of renewable energy has attracted increased attention. In recent years, solar heat pump heating technology that uses clean solar energy combined with high-efficiency heat pump units is the development direction of clean heating in winter in northern regions. However, the use of solar energy is intermittent and unstable. The low-valley electricity policy is a night-time electricity price policy. Heat pump heating has problems such as frosting and low efficiencies in cold northern regions. To solve these problems, an exergy analysis model of each component of a phase-change heat-storage coupled solar heat pump heating system was established. Exergy analysis was performed on each component of the system to determine the direction of optimization and improvement of the phase-change heat-storage coupled solar heat pump heating system. The results showed that optimizing the heating-end heat exchanger of the system can reduce the exergy loss of the system. When the phase-change heat-storage tank meets the heating demand, its volume should be reduced to lower the exergy loss of the tank heat dissipation. Air-type solar collectors can increase the income exergies of solar collectors.

## 1. Introduction

The use of solar heat pumps for combined heating has become a current development direction of clean heating. However, most of the current research is concentrated on the coupling of solar, photovoltaic/thermal energy, geothermal energy, or water source heat with heat pumps for heating research and economic analysis. Only a small number of studies have involved phase-change heat-storage materials. There are intermittency and stability issues associated with the use of solar energy. In the process of using solar energy, heat-storage equipment is required. Some European countries have taken the lead in building solar heat-storage heating projects. In 1956, Penrod [1,2] first proposed the combination of solar collectors and buried pipes for heating, pioneering a new method of solar thermal use.

Plytaria et al. [3] designed, simulated, and evaluated three different solar-assisted heat pump underfloor heating systems with and without phase-change materials. To improve the heat-storage capacity of the floor heating system and avoid the operation of the heat pump during the peak electricity consumption period, a phase-change material was used in the floor heating system.

Many scholars have carried out energy and exergy analyses on solar heat pump systems, comprehensively evaluated the energy use rate of each piece of equipment of the systems, and made judgments on the system energy-saving potential. Ozgener et al. [4] established an energy and exergy analysis model of a solar-ground-source heat pump system and conducted an experimental study on a system in Turkey. The results showed that the average COP of the system was about 3.14, and the average exergy efficiency reached 68.11%. Using thermodynamics analysis, Kuang et al. [5] proposed energy and exergy balance equations for the main equipment of a solar heat pump system, analyzed the system performance and energy-saving effect, and used it for the design and evaluation of the solar heat pump system. Wang [6] analyzed the energy use of each device of the conventionally operated solar heat pump system based on the first and second laws of thermodynamics, performed energy analysis of each link of the system, and presented the formula of exergy loss. Zhao et al. [7] derived an exergy balance equation of a circulation process in the heating mode of a solar-assisted heat pump and calculated the exergy losses of the main components of the system based on the experimental test data. The results showed that the exergy loss of the collector and compressor reached 31.84% and 14.89%, respectively. Cervantes et al. [8] conducted an experimental study on a solar heat pump system, determined its maximum exergy efficiency, and pointed out that the solar heat collector/evaporator had the largest exergy loss in the solar heat pump system.

The essence of the exergy analysis method is to combine the first law of thermodynamics with the second law of thermodynamics. To combine the quantity and quality of the energy, the conversion, transmission, and use processes of the effective energy in the device or equipment must be analyzed and revealed. This is the exergy balance calculation of the device or equipment, and the main thermodynamic index is the exergy efficiency [9]. This article introduces a composite system with phase-change heat-storage materials as the core, combination low-valley electricity use characteristics, solar thermal use characteristics, and heat pump systems.

A phase-change heat-storage coupled solar heat pump heating system was designed. The energy savings of the composite system and the characteristics of the exergy model analysis were qualitatively analyzed. Exergy analysis of each component of the system, the purpose is to find out the components with low energy efficiency in the system, and then re-optimize it in a targeted manner. Provide some reference for the optimization of solar heat pump heating system.

## 2. Materials and Methods

Guided by the first and second laws of thermodynamics, thermodynamic assumptions are established that can be quantitatively analyzed. Furthermore, an exergy analysis model for each component of the phase-change heat-storage coupled solar heat pump heating system was established. The energy use rate of each device was comprehensively analyzed to determine the energy-saving potential of the system. The system is mainly composed of solar collectors, phase-change heat-storage tank and heat pump system. The exergy will change with the change of the size of the solar heat collection, the phase-change heat-storage density and heat exchange efficiency, and the performance of the heat pump system. Therefore, it needs to be exergy analyzed. The compressor, evaporator, condenser, and throttle valve of the heat pump system are all key components that affect its performance and need to be exergy analyzed.

### 2.1. Phase-Change Heat-Storage Coupled Solar Heat Pump System

**Figure 1:**
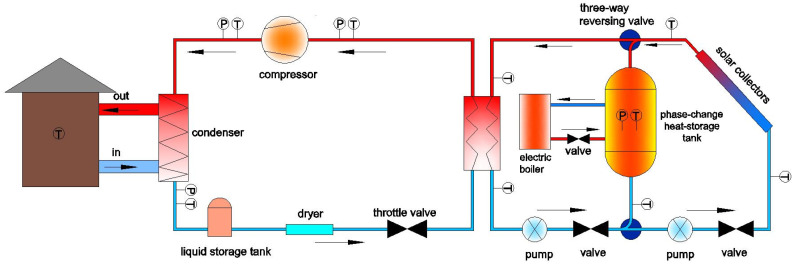
Schematic diagram of phase-change energy-storage coupled solar heat pump system. P—(Pressure Sensor), T—(Temperature Sensor).

The solar phase-change heat-storage evaporative heat pump system is a composite system that uses a phase-change heat-storage system as its core and is coupled with a solar system and a heat pump system to supply heat [10]. The system has two operating modes. When solar energy is sufficient, part of the heat is collected by a solar collector and used as a heat source when the heat pump is operating, and the other part is stored in the phase-change heat-storage tank, which is used when there is no solar energy. When the solar energy is insufficient or absent, low-valley electricity is used to heat the phase-change heat-storage material in the phase-change heat-storage tank to a set temperature at night, and the heat is used as a heat source of the heat pump. Low-valley electricity is also known as time-sharing electricity. Commercial electricity consumption at night is greatly reduced. To balance the electricity grid system, the electricity generation load cannot be significantly reduced, and the excess electricity generated by the electricity plant implements a periodic electricity consumption policy to achieve peak reduction and valley-filling of the electric grid. The low-valley electricity period is between 22:00–08:00, and the electricity price is half of the normal electricity price during the day. An advantage of this system is that the heat pump system does not need defrosting, which avoids frequent starting and stopping of the unit and prolongs the unit life. For areas with rich solar energy, the system can make full use of the solar energy and increase the proportion of solar heating. The phase-change heat-storage tank was filled with an aluminum ammonium dodecahydrate/stearic acid composite phase-change heat-storage material [11], and the phase-change temperature was 358.15 K, the latent heat of phase change is 246 kJ/kg. A schematic diagram of the system is shown in Figure 1.

### 2.2. Model Assumptions

By performing thermodynamic analysis on the actual cycle of the system, the entire system can be treated as a stable flow opening system. The amount of medium entering and exiting the flow opening system and each state parameter are stable and remain unchanged, and the following assumptions can be made [12].

(1) The fluid flow in the circulation is steady-state flow.
(2) The influence of kinetic energy and potential energy is neglected.
(3) The environment state is set to a constrained dead state.

In the actual cycle process, the process of producing exergy loss mainly includes the following:

(1) The non-isentropic compression process of the working fluid in the compressor.
(2) Condenser and evaporator heat transfer processes with limited temperature differences.
(3) The process in which the working fluid in the solar collector absorbs solar radiant heat.
(4) The heat absorption and release process of the working fluid in the phase-change heat-storage tank.
(5) The throttling process of the throttle valve.
(6) The mixing process of the working fluid in the suction pipe of the compressor.

### 2.3. Indoor Income Exergy

The water temperature of the supply and return water can be used to express the useful energy provided to the room by the phase-change heat-storage coupled solar heat pump heating system. The indoor income exergy is as follows [13]:

(1) $$E_{o}=M_{g}c_{p}\left(T_{g}-T_{h}-T_{a}ln\frac{T_{g}}{T_{h}}\right),$$

where $E_{o}$ is the indoor income exergy in kW, $M_{g}$ is mass flow of the heating medium in kg/s, $T_{g}$ is the heating supply temperature in K, $T_{h}$ is the return water temperature in K, $T_{a}$ is the outdoor temperature in K, $c_{p}$ is the specific heat capacity of water at constant pressure and the value is 4.2 kJ/(kg·K).

### 2.4. Exergy Equation of Solar Collectors

The exergy equation of solar collector [14,15] is as follows:

(2) $$E_{sun}=\psi A_{c}I\left(1-\frac{T_{a}}{T_{s}}\right)-M_{c}c_{p}\left(T_{co}-T_{ci}-T_{a}ln\frac{T_{co}}{T_{ci}}\right),$$

where $E_{sun}$. is the income exergy of the solar collector in kW, ψ is the absorption rate of the solar collector and the value is 85%, $A_{c}$ is the area of the solar collector in m^2^, I is the solar irradiation value in W/m^2^, $T_{a}$. is the outdoor temperature in K, $T_{s}$ is the center temperature of the solar collector in K, $M_{c}$ is the heat transfer medium flow rate of the solar collector in kg/s, and $T_{co}$ and $T_{ci}$ are the outlet and inlet temperatures of the solar collector in K, respectively.

### 2.5. Phase-Change Heat-Storage Tank Exergy Balance Equation

The phase-change heat-storage tank exergy balance equation can be expressed as follows [12]:

(3) $$E_{pcm}=F_{c}M_{c}c_{p}\left(T_{co}-T_{ci}-T_{a}ln\frac{T_{co}}{T_{ci}}\right)-Q_{pcm}\left(1-\frac{T_{a}}{T_{s}}\right)-F_{r}M_{g}c_{p}\left(T_{s}-T_{eo}-\;T_{a}ln\frac{T_{s}}{T_{eo}}\right)-F_{d2}\bar{F_{r}}M_{g}c_{p}\left(T_{sm}-T_{h}-T_{a}ln\frac{T_{sm}}{T_{h}}\right),$$

where $E_{pcm}$ is the exergy loss of the phase-change heat-storage tank in kW, $Q_{pcm}$ is the heat-storage rate of the phase-change heat-storage tank, defined as $m_{s}c_{pcm}\frac{dT_{sm}}{d\tau }$, *m*_*s*_ is the quality of the phase-change material in the heat-storage tank and the value is 2150 kg, $c_{pcm}$ is the constant pressure specific heat capacity of the phase-change material and the value is 1.68 kJ/(kg·K), $T_{sm}$ is the average temperature of the heat-storage tank, which is equivalent to $T_{s}$ in the hybrid heat-storage tank model, $T_{sm}=\left(t_{1}+t_{2}+\ldots +t_{m}\right)/m$ in K, $T_{eo}$ is the evaporator outlet temperature in K, and $F_{c}$, $F_{r}$, and $F_{d2}\;$ are control functions of the collectors, heat pumps, and phase-change heat-storage tank circulation pumps, respectively. The control functions take values of 1 when the component is operating, and 0 when it is not.

### 2.6. Exergy Analysis of Heat Pump System

(a) System input exergy $E_{H1}$ [8,16].

The input of the system is the input electric energy *P* of the compressor motor, which can be obtained by actual measurements. The measured electrical energy input to the motor is the power value. For calculation convenience, this needs to be converted to a work per unit mass. The mass flow rate of the working fluid needs to be calculated, and the heat pump heating capacity $Q_{H}$ can be measured first. The mass flow of the working fluid can be calculated as follows:

(4) $$M=\frac{Q_{H}}{h_{1}-h_{4}}$$

where M is the mass flow of the working fluid of the heat pump system in kg/s, $h_{1}$ is the working fluid evaporator outlet specific enthalpy in J/kg, and $h_{4}$ is the working fluid evaporator inlet specific enthalpy in J/kg.

The motor input work per unit mass is as follows:

(5) $$W_{G}=Pm/M.$$

where m is the mass of the working fluid of the heat pump system and the value is 2.6 kg.

The input exergy of the system is as follows:

(6) $$E_{H1}=W_{G}.$$

(b) Exergy loss of motor.

The formula of the motor exergy loss is as follows:

(7) $$D_{d}=E_{H1}\left(1-\eta_{d}\right),$$

where $D_{d}$ is the exergy loss of the motor in kW, and $\eta_{d}$ is the motor efficiency in %.

(c) Compressor exergy loss.

**Figure 2:**
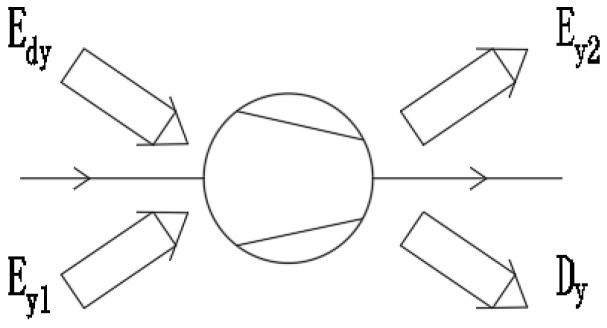
Schematic diagram of compressor exergy balance model.

The schematic diagram of the compressor exergy balance model is shown in Figure 2. The exergy entering the compressor includes the compressor input exergy $E_{dy}$ and the compressor inlet working fluid exergy $E_{Y1}$. The compressor output exergy includes the compressor outlet working fluid exergy $E_{Y2}$ and the compressor exergy loss $D_{y}$.

The exergy entering the compressor includes the input exergy and the compressor inlet working fluid exergy:

(8) $$E_{dy}=E_{H1}-D_{d},$$

(9) $$E_{Y1}=h_{1}-T_{a}S_{1},$$

where $E_{dy}$ is the compressor input exergy in kW, $E_{Y1}$ is the compressor inlet working fluid exergy in kW, and $S_{1}$ is the compressor inlet working fluid entropy in kW/K.

The compressor output exergy includes the compressor outlet working fluid exergy and the compressor exergy loss:

(10) $$E_{Y2}=h_{2}-T_{a}S_{2},$$

(11) $$D_{y}=E_{dy}+E_{Y1}-E_{Y2},$$

where $E_{Y2}$ is the compressor export working fluid exergy in kW, $D_{y}$ is the compressor exergy loss in kW, $h_{2}$ is the compressor outlet working fluid specific enthalpy in J/kg, and $S_{2}$ is the compressor outlet working fluid entropy in kW/K.

In the heat pump system, the compressor exergy loss accounts for a large proportion of the exergy loss. To reduce this loss, a compressor with a high isentropic efficiency can be used.

(d) Exergy loss of throttle valve.

The exergy loss of the throttle valve is the difference between the exergies of the inlet and outlet throttle valves:

(12) $$D_{J}=E_{J1}-E_{J2},$$

(13) $$E_{J1}=h_{3}-T_{a}S_{3},$$

(14) $$E_{J2}=h_{4}-T_{a}S_{4},$$

where $D_{J}$ is the throttle valve exergy loss in kW, $E_{J1}$ is the throttle valve inlet working fluid exergy in kW, $E_{J2}$ is the throttle valve outlet working fluid exergy in kW, $h_{3}$ is the throttle valve inlet working fluid specific enthalpy in J/kg, $h_{4}$ is the throttle valve outlet working fluid enthalpy in kW, $S_{3}$ is the working fluid entropy of the throttle value inlet in kW/K, and $S_{4}$ is the throttle valve outlet working fluid entropy in kW/K.

(e) Condenser exergy loss [7].

**Figure 3:**
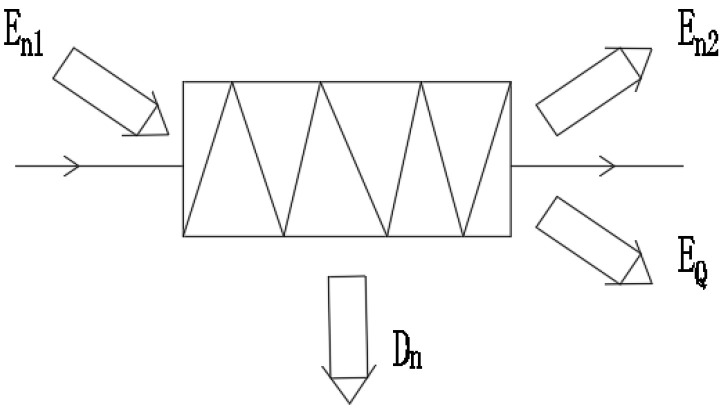
Schematic diagram of the condenser tritium balance model.

Figure 3 is a schematic diagram of the condenser exergy balance model. The exergy entering the condenser is the condenser inlet working fluid exergy $E_{n1}$, and the condenser output exergy includes the condenser outlet working fluid exergy $E_{n2}$, the system heating exergy $E_{Q}$, and the condenser exergy loss $D_{n}$.

The formula of the condenser exergy loss is as follows:

(15) $$D_{n}=E_{n1}-E_{n2}-E_{Q},$$

(16) $$E_{Q}=\frac{T_{h}-T_{a}}{T_{a}}\left(h_{1}-h_{2}\right),$$

where $E_{n1}$ is condenser inlet working fluid exergy in kW, $E_{n2}$ is the condenser outlet working fluid exergy in kW, $E_{Q}$ is the system heating exergy in kW, and $T_{h}$ is the condenser medium temperature in K.

(f) Evaporator exergy loss.

The formula for calculating the exergy loss of the evaporator is as follows:

(17) $$D_{f}=E_{f1}-E_{f2},$$

where $D_{f}$ is the evaporator exergy loss in kW, $E_{f1}$ is the evaporator inlet working fluid exergy in kW, and $E_{f2}$ is the evaporator outlet working fluid exergy in kW.

(g) Heat pump system exergy efficiency $\eta_{ep}$.

The formula for the heat pump system exergy efficiency is as follows:

(18) $$\eta_{ep}=1-\frac{\sum D}{\sum E_{H1}},$$

(19) $$\sum D=D_{d}+D_{y}+D_{n}+D_{J}+D_{f}.$$

### 2.7. System Evaluation Indicators Based on Exergy Analysis

In the phase-change heat-storage coupled solar heat pump heating system, the system investment exergy includes the exergy obtained by the solar collector and the exergy introduced by the input of conventional energy (electric energy). This research defines the exergy brought about by conventional energy input as the cost exergy.

The definition of the system exergy and cost exergy efficiencies are as follows [17]:

(20) $$\eta_{0}=\frac{E_{o}+E_{sun}}{E_{d}},$$

(21) $$\eta_{ex}=\frac{E_{o}}{E_{d}},$$

where $\eta_{0}$ is the system exergy efficiency, $\eta_{ex}$ is the cost exergy efficiency, $E_{d}$ is the cost exergy in kW, $E_{sun}$ is the income exergy of the solar collector in kW, and $E_{o}$ is the indoor income exergy in kW.

The cost can be expressed as follows:

(22) $$E_{d}=E_{d1}+E_{d2}+E_{d3}+E_{d4}+E_{dp}+E_{de},$$

where $E_{d1}$ is the cost exergy consumed by the solar collector circulating pump in kW, $E_{d2}$ is the cost exergy consumed by the phase-change heat-storage tank circulating pump in kW, $E_{d3}$ is the cost exergy consumed by the heater heating circulating pump in kW, $E_{d4}$ is the cost exergy consumed by the user-side heating circulating pump in kW, $E_{dp}$ is the cost exergy consumed by the heat pump system in kW, and $E_{de}$ is the cost exergy consumed by the electric boiler heating in kW.

The cost exergy efficiency of this system is as follows:

(23) $$\eta_{ex}=\frac{E_{o}}{E_{d1}+E_{d2}+E_{d3}+E_{d4}+E_{dp}+E_{de}}.$$

## 3. Results and Discussion

An oil depot office in Tongliao was selected as the application object of the phase-change heat-storage coupled solar heat pump heating system. The heating area of the building was 150 m^2^, and the heating load index of the building was 91.6 W/m^2^. The average outdoor temperature was 263.45 K, and the average indoor design ambient temperature was 291.15–295.15 K. The phase-change heat-storage tank was filled with an aluminum ammonium dodecahydrate/stearic acid composite phase-change heat-storage material, and the phase-change Melting temperature was 358.15 K. The filling rate was 85%, and the volume of the phase-change heat-storage tank was 2.5 m^3^.

**Figure 4:**
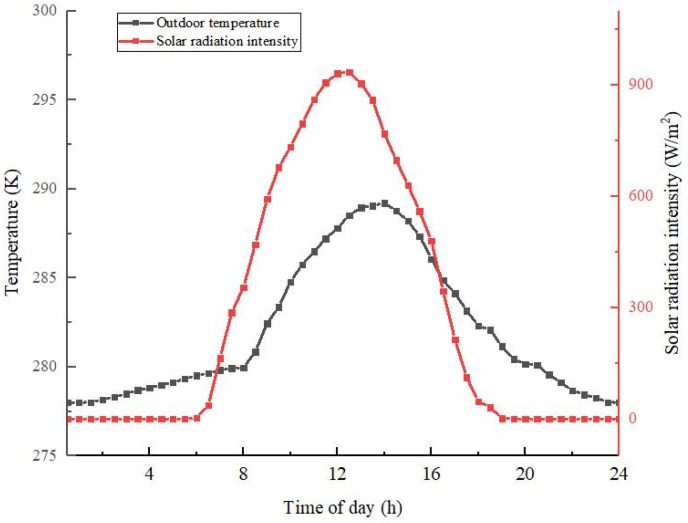
Typical outdoor temperature and solar radiation intensity.

A typical day was selected during which the irradiance per unit solar collector area was 20.5 MJ/m^2^, the maximum solar irradiance was 935 W/m^2^, and the required solar collector area was 48 m^2^. The heat consumption of the building on that day was 296 MJ, and the total irradiance on the lighting surface of the solar collector was 162 MJ. The electric heater power was selected as 22 kW. Figure 4 shows the variation of the outdoor temperature and solar radiation intensity for a typical day in Tongliao City, Inner Mongolia.

### 3.1. Analysis of Influence of Area of Solar Collector on Exergy Efficiency of System

Exergy analysis of the solar collector was performed. When the solar radiation intensity and area of the solar collector were fixed values, the smaller the mass flow of the heat exchange medium of the solar collector was, the greater the profit of the solar collector became. Therefore, the designed air-type solar collector could increase the revenue of the solar collector. The Spiral Double-pass Direct-current (SDDC) Structure was applied to the vacuum tube of air-type solar energy collector [18]. In addition, the phase-change heat-storage rod in the vacuum tube of the air-type solar collector could store more solar energy, which could further increase the revenue of the solar collector.

**Figure 5:**
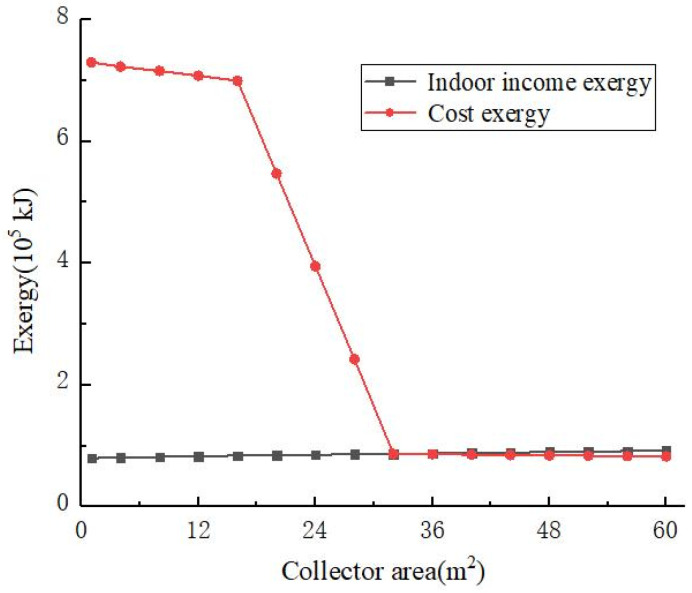
Variations of the cost and indoor income exergies with the area of the solar collector.

Figure 5 shows the variations of the system cost exergy and the indoor income exergy with the area of the solar collector. The indoor revenue exergy increased slowly with the increase in the solar collector area. The cost of the system first decreased with the increase in the area of the solar collector, and the cost of the system decreased rapidly when the area of the solar collector was 16–32 m^2^. This was because as the area of the solar collector increased, the exergy obtained by the system increased, and the cost of the system decreased. According to Equation (2), when the area of the solar collector increased to about 16 m^2^, the center temperature of the solar collector no longer increased with the increase in the area of the solar collector. Therefore, at this time, the system cost exergy curve underwent a sudden downward trend. When the area of the solar collector increased to about 32 m^2^, the system cost became stable and no longer changed with the increase in the area of the solar collector.

**Figure 6:**
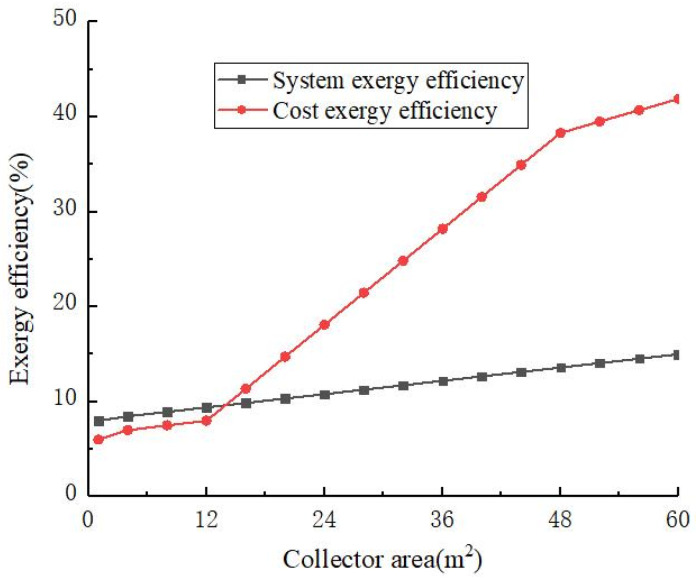
The exergy efficiency changes with the area of solar collectors.

Figure 6 shows the exergy efficiency changes with the area of solar collectors. Both increased with the increase in the solar collector area. When the area of the solar collector was 48.2 m^2^, the exergy efficiency reached 38.3%. Since then, the growth rate of the cost exergy efficiency as the area of the solar collector increased began to diminish. The exergy efficiency of the system exhibited an upward trend with the increase in the area of the solar collector, and the fluctuations were small. Since the area of each solar collector was a fixed value of 4 m^2^, the number of solar collectors selected in the design was 12, with a total area of 48 m^2^. It can be seen that the area of the solar collector of the system is not as large as possible, and too many collectors cannot continuously improve the use rate of solar energy.

### 3.2. Influence of Phase-Change Heat-Storage Tank Volume on Exergy Efficiency of System

**Figure 7:**
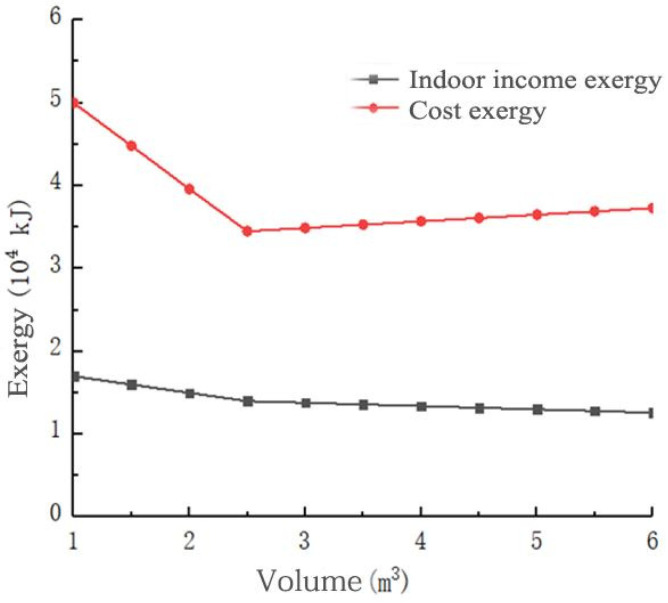
Variations of the cost and indoor income exergies with the volume of the phase-change heat-storage tank.

Figure 7 shows the variations of the cost exergy of the system input and the indoor income exergy with the volume of the phase-change heat-storage tank. The indoor income exergy decreased slowly as the volume of the phase-change heat-storage tank increased. As the volume of the phase-change heat-storage tank increased, the cost of the system input decreased first and then slowly increased. When the volume of the phase-change heat-storage tank was 2.6 m^3^, the minimum cost exergy was 3.4 × 10^4^ kJ. Equations (2), (3) and (22) show that as the volume of the phase-change heat-storage tank increased, the amount of heat collected by the solar collector and stored in the phase-change heat-storage tank increased. The exergy gained by the system gradually increased, and the exergy cost of the system gradually decreased. When the volume of the phase-change heat-storage tank was 2.6 m^3^, the heat collected by the solar collector could be completely stored in the phase-change heat-storage tank, and the exergy obtained by the system reached the maximum value. Therefore, the system cost exergy value was the smallest. When the phase-change heat-storage tank volume was greater than 2.6 m^3^, as the phase-change heat-storage tank volume increased, the system cost exergy increased.

**Figure 8:**
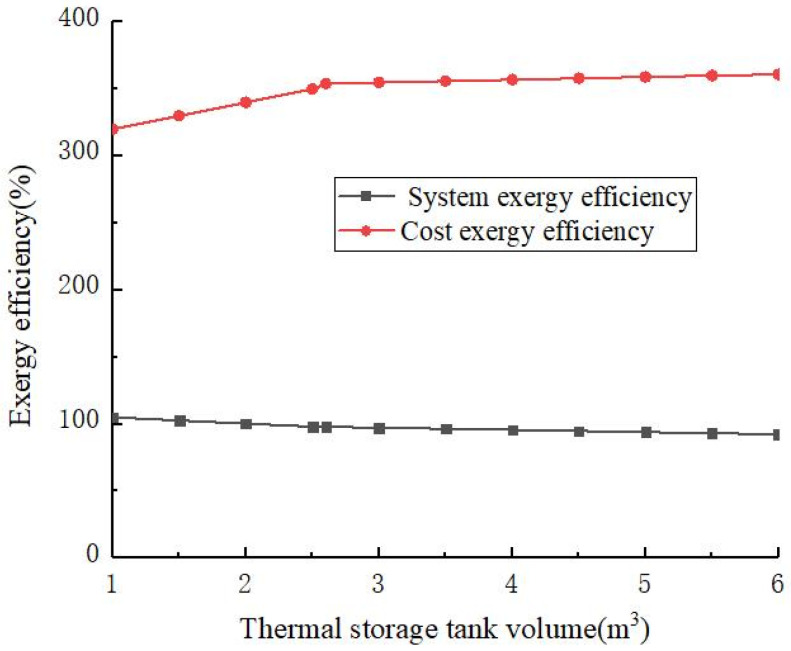
The exergy efficiency changes with the volume of phase-change heat-storage tank.

Figure 8 shows the exergy efficiency changes with the volume of phase-change heat-storage tank. As the volume of the phase-change heat-storage tank increased, the exergy efficiency of the system slowly decreased. When the volume of the phase-change heat-storage tank was less than 2.6 m^3^, the cost efficiency increased with the increase in the volume of the phase-change heat-storage tank. When the volume of the phase-change heat-storage tank was greater than 2.6 m^3^, the cost exergy efficiency increased slowly with the increase in the volume of the phase-change heat-storage tank. Based on the results shown in Figure 7 and Figure 8, the optimal phase-change heat-storage tank volume was 2.6 m^3^.

It can be seen from Figure 7 and Figure 8, the volume of the phase-change heat-storage tank should first meet the requirements of heat storage, and secondly, the increase in the cost exergy and cost exergy efficiency of the phase-change heat-storage tank should be considered, i.e., the heat preservation cost and heat exchange efficiency of the phase-change heat-storage tank.

### 3.3. Influence of Other Factors on Efficiency of System

Through the analysis of the exergy balance equation, it was determined that the indoor income exergy $E_{o}$ of the system was a fixed value, because the indoor temperature was a fixed value. If the mass flow and temperature of the heating medium water remained unchanged, increasing the heat exchange efficiency of the heat exchanger at the end of the heating could further save energy for the system. The mass flow of the heat exchange medium (water) at the heating end was inversely proportional to the temperature difference between the supply and return water. When the set indoor heat load was a constant value, the temperature difference between the supply and return water was also a constant value. Increasing the mass flow of the heat exchange medium (water) at the heating end within a reasonable range could improve the heat exchange efficiency of the heat exchanger at the heating end, therefore increasing the indoor revenue.

The exergy loss during the throttling process in the capillary tube and the mixing process at the suction pipe of the compressor was relatively small. To reduce the exergy loss in the throttling link, the steam regenerative cycle method could be adopted; that is, an auxiliary heat exchanger could be added. The low-temperature gaseous working fluid from the evaporator would pass through the auxiliary heat exchanger before entering the compressor to exchange heat with the saturated working fluid from the condenser. As a result, the saturated working fluid temperature would be lowered before throttling, therefore reducing the exergy loss [14].

In addition, it is possible to further reduce the exergy loss and improve the energy efficiency by increasing the flow rate of the working fluid, reducing the irreversible throttling caused by grease and dirt and optimizing the design plan. Using an electronic expansion valve to replace the traditional capillary tube can further improve the performance of the system. The electronic expansion valve collects and calculates the parameters through the sensor and keeps the evaporator superheat within a certain range by opening and closing the drive valve, which is especially suitable for a heat pump unit with complex and changeable operating conditions [7]. Therefore, this system used an electronic expansion valve instead of a capillary tube.

For the same heat supply, the heat pump efficiency was always greater than 1, while the phase-change heat-storage tank heating efficiency was less than 1. The exergy loss of the heat pump heating system was much smaller than that of the phase-change heat-storage tank heating system. When the phase-change heat-storage tank system was used as the main heating system, the required phase-change heat-storage tank volume was larger. The cost of the electric heating was greater, and the cost exergy efficiency of the system was lower. Therefore, when conditions permit, the heat pump system should be combined for heating as much as possible.

## 4. Conclusions

An oil depot office in Tongliao City was used as the application object of the phase-change heat-storage coupled solar heat pump heating system. The exergy balance of each component of the system was analyzed, and the cost exergy, indoor revenue exergy, system exergy efficiency, and cost exergy efficiency were determined. The area of the solar collector, the volume of the phase-change heat-storage tank, and the heat exchanger at the heating end of the phase-change heat-storage coupled solar heat pump heating system were optimized. The conclusion will help to provide certain theoretical guidance for the optimization of the solar collector area, phase-change storage tank volume, heat pump system, and user-side heat exchanger of related systems. The results are summarized as follows:

(1) When the area of the solar collector was 48.2 m^2^, the cost exergy was small, and the cost exergy efficiency reached 38.3%. According to the integrity characteristics of the solar collector, a solar collector area of 48 m^2^ was optimal. Air-type solar collectors can increase the profitability of solar collectors, so this design is beneficial.
(2) When the volume of the phase-change heat-storage tank was 2.6 m^3^, the cost of the system input reached the lowest value of 3.4 × 10^5^ kJ. The volume of the optimized phase-change heat-storage tank changed from 2.5 to 2.6 m^3^.
(3) The heat exchange efficiency of the heat exchanger at the end of heating should be improved, as it can reduce the exergy loss of the system. When conditions permit, this heat exchanger should be combined with a heat pump system for heating. The capillary tube in the heat pump system can be replaced with an electronic expansion valve, which can reduce the exergy loss of the heat pump system.

## Data Availability

Not applicable.
